# Use of allogeneic platelet-rich plasma for the treatment of autoimmune ocular surface disorders: case series

**DOI:** 10.3389/fopht.2023.1215848

**Published:** 2023-10-05

**Authors:** Maura Mancini, Elisa Imelde Postorino, Ludovica Gargiulo, Pasquale Aragona

**Affiliations:** Department of Biomedical Sciences, Ophthalmology Clinic, University of Messina, Messina, Italy

**Keywords:** dry eye disease (DED), allogeneic platelet rich plasma, growth factors, Sjogren’s syndrome, autoimmune ocular surface disorders

## Abstract

**Purpose:**

To assess the effectiveness of topical allogeneic platelet-rich plasma (PRP) eye drops for the treatment of symptoms and clinical signs in patients with severe dry eye disease as a secondary condition caused by Sjögren’s syndrome (SS).

**Design:**

Case series and literature review.

**Methods:**

Six eyes from three consecutive patients with severe dry eye from SS were evaluated. The eyes were treated with allogeneic topical PRP eye drops, with one drop applied six times daily for 3 months. A post-treatment follow-up evaluation was conducted 3 months after treatment suspension. We evaluated subjective symptoms, visual acuity, tear breakup time, the results of Schirmer’s I test, fluorescein corneal and conjunctival staining, and corneal sensitivity.

**Results:**

The symptoms and visual acuity improved significantly in all patients. There was a significant improvement in corneal sensitivity and a decrease or disappearance of fluorescein corneal staining.

**Conclusion:**

The treatment with allogenic PRP eye drops of patients with SS-related severe dry eye disease has proven to be very effective, with an improvement in symptoms and main clinical signs.

## Introduction

Sjögren’s syndrome (SS) is one of the main causes of severe dry eye disease (DED). DED affects about 8% of the over 108 million Americans over the age of 50 years; about 1 out of 10 of these DED patients have underlying SS (1).

SS manifests as a chronic and debilitating inflammation, which is mediated by the production of autoantibodies and lymphocytic infiltration, and, ultimately, causes permanent destruction of the exocrine glands, resulting in “sicca” symptoms, such as dry eyes and dry mouth (2).

The damage to the ocular surface caused by SS can range from instances of superficial punctate keratitis of varying levels of severity to, if not diagnosed and treated accurately, more severe cases with corneal ulcers and scars that can significantly impair visual function and even cause irreversible blindness.

Today, the use of blood-based eye drops as a therapy for various diseases of the eye surface has become increasingly popular in ophthalmic practice. Their use is based on the promotion of cell proliferation and migration owing to the presence of metabolically active substances, in particular growth factors (3).

The use of blood products as a treatment for pathologies of the eye surface was developed over 40 years ago by administering autologous serum-based eye drops to patients with corneal chemical damage (4) and, 10 years later, to patients with DED related to SS (5).

At present, the use of these substances has improved and has been applied in severe cases of ocular surface damage in which conventional treatments are not sufficient to restore the homeostasis of this delicate system.

Compared with more traditional therapies, blood-derived eye drops contain several biochemical constituents that function in a way that is more similar to natural tears (6, 7). Their effect on the growth and proliferation of corneal epithelial cells has been demonstrated in *in vitro* and *in vivo* studies (8–10).

Platelet-rich plasma (PRP), obtained from total non-clotted blood, is very rich in platelets and growth factors (GFs). GFs are biological mediators that promote cell proliferation by binding to specific cell surface receptors (11). There are many growth factors, and each of them has been reported to accelerate different aspects of wound healing (12, 13).

Platelets are known to secrete some of these factors from alpha granules. To date, the following GFs have been found to derive from platelets: platelet-derived angiogenesis factor, platelet-derived growth factor, platelet-derived epidermal growth factor, and platelet-derived factor IV (14–16).

PRP has been used for over a decade in several clinical areas such as orthopedics (17) and maxillofacial surgery (18, 19).

Since PRP eye drops were added to the suite of available therapeutical interventions in ophthalmology, they have shown very promising results (20, 21), improving cornea regeneration in case of keratitis, reducing inflammation, accelerating and stimulating the wound healing processes, and producing a lubricating effect (22).

In patients with SS, eye drops of allogeneic origin must be used, otherwise the presence of autoantibodies or cytokines in the autologous preparations can cause increased inflammation and damage to the ocular surface.

We report on a consecutive, non-randomized case series, conducted to prospectively assess the efficacy of topical allogenic PRP administration for the treatment of clinical symptoms and signs in patients with severe DED as a secondary condition caused by SS.

## Materials and methods

### Patients

Six eyes of three patients with severe DED due to SS were prospectively investigated at the Ocular Surface Diseases Unit of the Institute of Ophthalmology, University of Messina, Messina, Italy.

The patients had severe persistent epithelial defects (PEDs) not responsive to conventional treatments (e.g., artificial tears and topical anti-inflammatories) with a significant reduction in corneal sensitivity.

In addition to their usual therapy, patients were treated with allogeneic PRP eye drops prepared at the Transfusion Service of the University Hospital “G. Martino” of Messina. The prescribed regimen for the eye drops was one drop six times a day for 3 months, and outcomes were evaluated at a follow-up visit 3 months after the suspension of the treatment.

The patients signed an informed consent form for the use of personal data and images from the study.

### Measures

For each patient, the following clinical parameters were evaluated before and after the treatment: subjective symptoms on the Ocular Surface Disease Index© (OSDI©) scale, best corrected visual acuity (BCVA; Snellen) test, tear breakup time (TBUT) test, corneal and conjunctival staining with fluorescein, and Schirmer’s I test, and corneal sensitivity (with the Cochet–Bonnet aesthesiometer).

The classification criteria developed by the Sjögren’s International Collaborative Clinical Alliance (SICCA) is based on a quantitative assessment of conjunctival surface damage. It has been used for the assessment and staging of SICCA keratoconjunctivitis, using fluorescein staining for the cornea assessment and lissamine green for the conjunctiva.

The score on the SICCA Ocular Staining Score (OSS) grading system is obtained by summing the fluorescein score with the lissamine green score, with a maximum value of 12 for each eye. The OSS scores above three are considered pathological and indicative of a DED diagnosis (23).

### Allogeneic platelet-rich plasma preparation

Platelet-rich plasma is characterized by a platelet concentration of over 1 × 106/mL. To obtain PRP, 30 mL of venous blood, from donors with the same blood type as the patient, is drawn in tubes with 3.2% sodium citrate to prevent platelet activation prior to its use. A first centrifugation at a relatively low speed (i.e., 10 minutes of a soft spin from 200 g to 600 g) separates the whole blood into three layers: an upper layer that contains mostly platelets and white blood cells (WBCs); an intermediate thin layer of a whitish color, called buffy coat (BC), which is rich in WBCs; and a bottom layer that consists of mostly red blood cells (RBCs). The upper layer and superficial BC are transferred into another sterile tube for a second centrifugation step at higher speeds (i.e., 10 minutes of a hard spin at 2,300 g) to concentrate the platelets. The upper two-thirds of the volume, called platelet-poor plasma (PPP), is discarded, whereas the lower one-third (5 mL of plasma) is homogenized by gently shaking the tube to create PRP. The product can be used either undiluted or diluted in balanced salt solution (BSS), sealed in vials or bottles, and stored in the refrigerator at 4°C for a maximum of 1 week or at −20°C for longer periods (3).

## Case 1

Case 1 was a 47-year-old woman, presenting with an undifferentiated connective tissue disease for 20 years, Hashimoto’s thyroiditis for 15 years, and scleroderma for 4 years, and receiving systemic therapy with 200 mg/day of hydroxychloroquine (Plaquenil®; Sanofi Italy, Milan, Italy) for 20 years. She presented to our clinic citing intense photophobia and burning and foreign body sensations. The clinical evaluation found significant impairment of the ocular surface, with reduced tear film stability, mucous plaques in the tear film, and persistent epithelial defects (PEDs). Furthermore, Case 1 was also found to have conjunctival epithelium alterations with conjunctival folds.

The initially prescribed therapy comprised warm compresses; preservative-free tear substitutes containing 0.2% sodium hyaluronate (Hyalistil BIO PF; SIFI, Aci Sant’Antonio, Italy); and, if there was a severe exacerbation of symptoms, corticosteroid treatment with either 0.3% hydrocortisone or 0.1% dexamethasone, depending on the clinical severity.

Given the frequent clinical exacerbations and the risks linked to corneal hypoesthesia, we decided, after 6 months of unsuccessful treatment, to add allogeneic PRP eye drops administered six times a day for 3 months.

**Figure 1:**
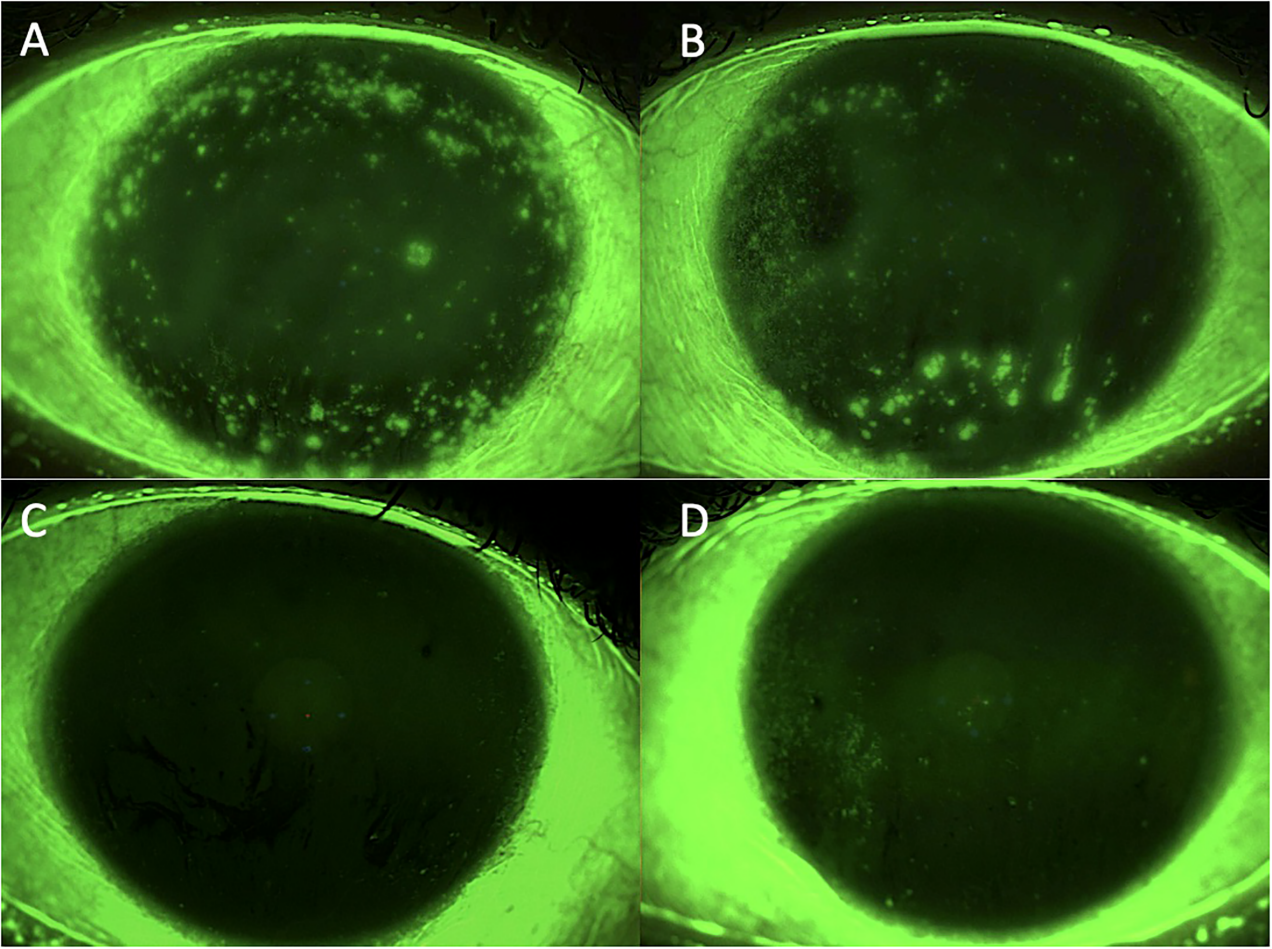
Case 1: effect of the treatment with platelet-rich plasma eye drops on non-healing persistent epithelial defects (PEDs). **(A)** right eye before treatment; **(B)** left eye before treatment; **(C)** right eye after 3 months of treatment; and **(D)** left eye after 3 months of treatment.

After 3 months of treatment the evaluated symptoms and clinical parameters showed a significant improvement, as shown in **Table 1** and **Figure 1**. Furthermore, 3 months after PRP eye drops treatment suspension, the clinical features remained stable without any further need for corticosteroid treatment.

**Table 1:** The clinical features before and after treatment with platelet-rich plasma eye drops.

	OSDI			BCVA			TBUT (s)			SCHIRMER (mm/min)			OSS GRADING			COCHET– BONNET (cm)			
PRE TREATMENT	RE		LE	RE		LE	RE	LE		RE	LE		RE	LE		RE		LE	
Case 1	53		44	8/10		9/10	1	2		1/5	2/5		8	7		2		3	
Case 2	88		92	3/10		2/10	2	1		1/5	0/5		12	12		1		0	
Case 3	69		74	8/10		5/10	3	1		3	2		11	12		2		2	

	OSDI			BCVA			TBUT (s)			SCHIRMER (mm/min)			OSS GRADING			COCHET– BONNET (cm)			
POST TREATMENT	RE		LE	RE		LE	RE	LE		RE	LE		RE	LE		RE		LE	
Case 1	33		30	10/10		10/10	3	3		2	2		3	4		4		4	
Case 2	40		44	7/10		5/10	3	3		2	2		5	5		3		2	
Case 3	34		37	9/10		9/10	3	3		3	3		4	4		3		3	

## Case 2

Case 2 was a 70-year-old woman presenting with rheumatoid arthritis for the last 20 years and in systemic therapy with 200 mg/day of hydroxychloroquine (Sanofi Italy). This was a challenging case, as the woman had undergone radial keratotomy with 12 incisions in the right eye and 16 in the left eye for myopic correction 35 years ago. The patient was also being treated with hypotensive drops [0.1% timolol quaque die (QD), 0.2% brimonidine bis in die (BID)] for glaucoma, beginning 10 years ago. All the clinical features listed above had led to corneoconjunctival lesions, resulting in visual acuity and corneal sensitivity impairment. **Table 1** presents the patient’s clinical features.

The initially prescribed therapy comprised warm compresses, preservative-free tear substitutes containing 0.2% sodium hyaluronate (Hyalistil BIO PF; SIFI), and 0.1% cyclosporine A eye drops (Ikervis®; Santen Pharmaceutical Co., Ltd., Milan, Italy), with one drop administered four times a day.

As the patient’s clinical features were not improving, despite the treatment and with a high risk of neurotrophic ulcer development, allogeneic PRP eye drops administered six times a day for 3 months were added to the treatment.

**Figure 2:**
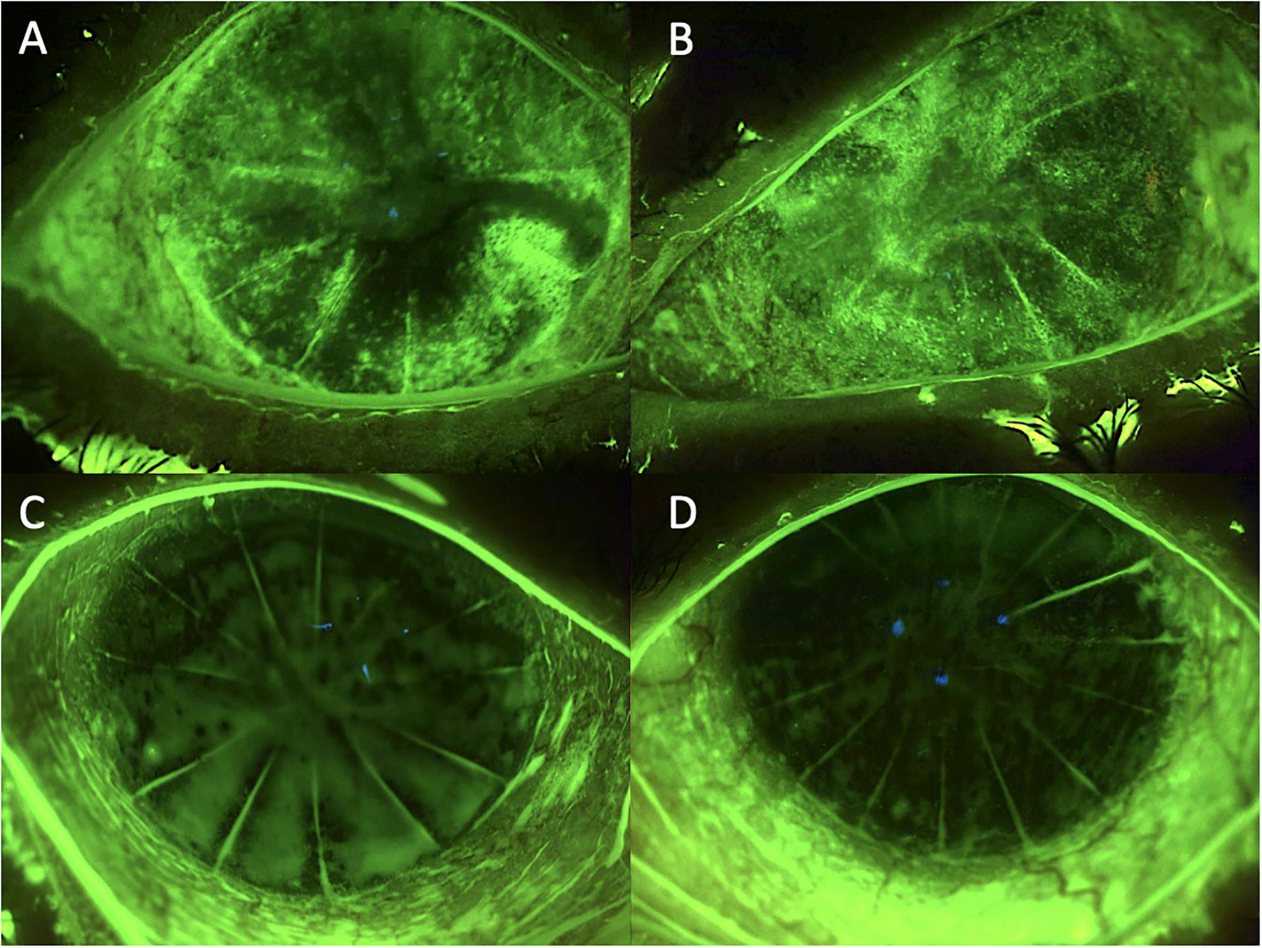
Case 2: effect of the treatment with platelet-rich plasma eye drops on non-healing persistent epithelial defects (PEDs) in a patient who previously underwent radial keratotomy. **(A)** right eye before treatment; **(B)** left eye before treatment; **(C)** right eye after 3 months of treatment; and **(D)** left eye after 3 months of treatment.

After 3 months of treatment with PRP eye drops, the evaluated symptoms and clinical parameters showed a significant improvement. The results are shown in **Table 1** and **Figure 2**.

## Case 3

A 63-year-old female patient was admitted to our unit with a history of primary SS since 2009.

At the clinical evaluation, the patient presented with severe corneoconjunctival damage.

**Figure 3:**
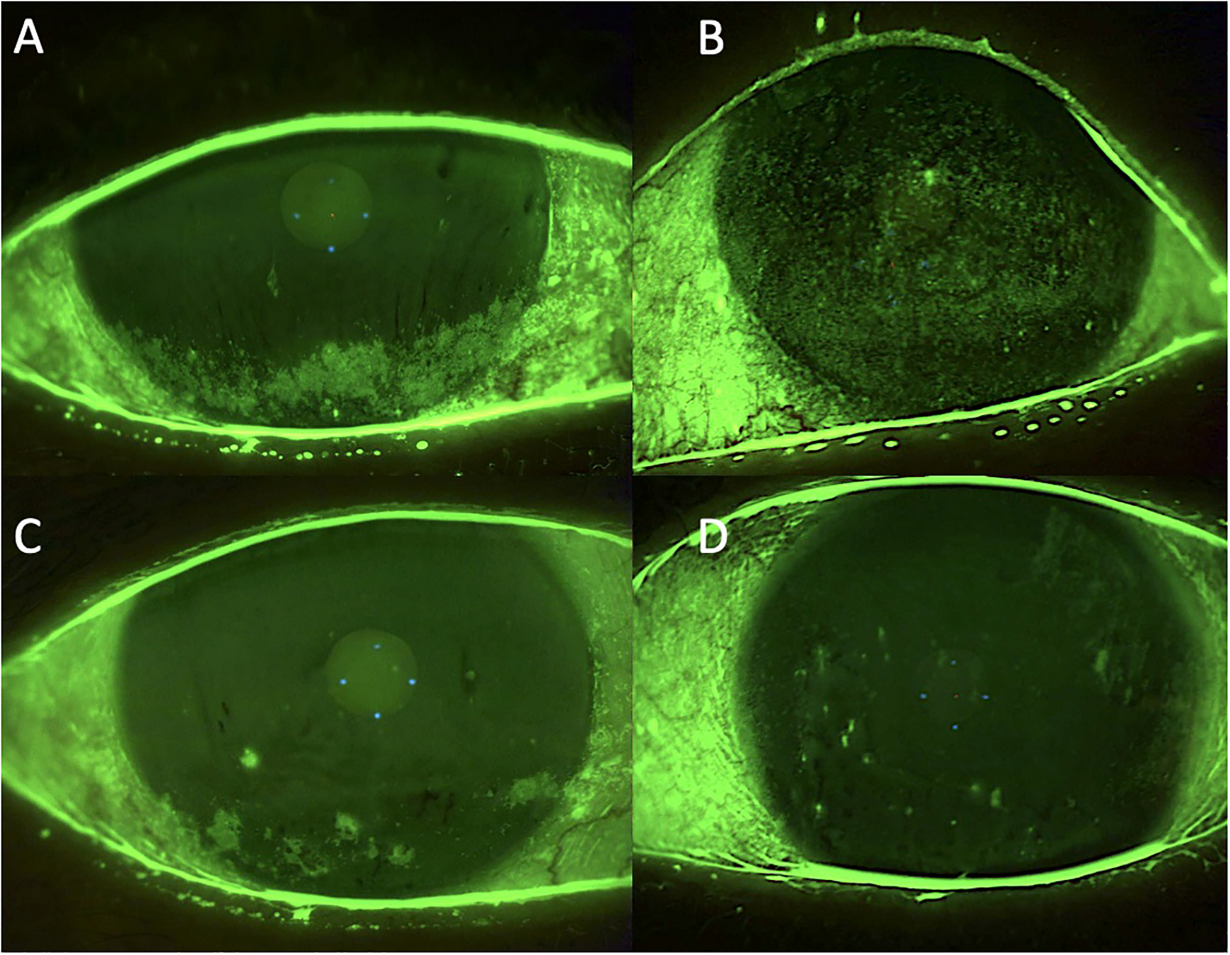
Case 3: effect of the treatment with platelet-rich plasma eye drops on non-healing persistent epithelial defects (PEDs). **(A)** right eye before treatment; **(B)** left eye before treatment; **(C)** right eye after 3 months of treatment; and **(D)** left eye after 3 months of treatment.

**Table 1** shows the patient’s clinical features. The initially prescribed therapy comprised warm compresses, preservative-free tear substitutes, and, if there was a severe exacerbation of symptoms, corticosteroid treatment with either 0.3% hydrocortisone or 0.1% dexamethasone 0.1%, depending on the clinical severity. Allogeneic PRP eye drops administered six times a day for 3 months were added to the therapy. After 3 months of treatment with PRP eye drops, the evaluated symptoms and clinical parameters showed a significant improvement. The results are shown in **Table 1** and **Figure 3**.

At the 3-month follow-up, the clinical evaluation showed a stable condition.

## Discussion

DED treatment in patients with SS, unlike in patients with other forms of DED, must address severe inflammatory components and more complex clinical presentations. The first line of treatment for DED in SS is preservative-free tear substitutes and anti-inflammatory therapy, alongside the use of topical corticosteroids or immunomodulators.

The use of blood products as a treatment for ocular surface diseases has greatly improved in recent years and can now be applied in severe cases that are unresponsive to conventional treatments.

These blood products consist of eye drops derived either from the patient’s own peripheral blood (an autologous source) or from a donor (an allogeneic source, taken from adult or umbilical cord blood) and prepared in the form of a serum comprising PRP, plasma rich in growth factors (PRGF), and platelet lysate (PL) (24).

Several issues surrounding PRP eye drops are still under debate, relating both to the lack of a standardized protocol for the preparation and storage of the eye drops and to the absence of a consensus on the clinical strategy necessary to achieve the best results, including the optimal clinical outcomes, patient stratification, length of treatment, and grounds for repeated treatment (3).

Although serum eye drops (SEDs) of autologous origin are still the most manufactured product for DED treatment, an increasing number of centers worldwide are now producing SEDs from allogeneic blood donors (25, 26). Moreover, some authors have reported that the peripheral serum of patients with SS and ocular graft-versus-host disease (oGVHD), which represent two causes of severe DED, might contain high concentrations of pro-inflammatory mediators potentially harmful to the ocular surface (27).

Compared with serum-based eye drops, platelet-based eye drops contain more growth factors and adhesion molecules and fewer pro-inflammatory cytokines (28).

Platelets play a main role in the wound healing process, as they have high concentrations of GFs and cytokines contained in their α-granules, such as platelet-derived growth factor (PDGF), transforming growth factor beta (TGF-β), and platelet factor IV (29). PRP acts by stimulating the release of PDGF, which is the first growth factor (GF) involved in wound healing, and of TGF-β. PDGF triggers the increase of activated macrophages along with the development of new blood vessels. TGF-β can induce chemotaxis and control epithelial proliferation, maintaining cells in an undifferentiated state. Furthermore, other important factors include epidermal growth factor (EGF), which accelerates corneal epithelial proliferation, and vascular endothelial growth factor (VEGF) and fibroblast growth factor 2 (FGF-2), which play a role in the angiogenesis process (30).

A 2023 study highlighted the superiority of PRP in relieving the symptoms of DED in patients with primary Sjogren’s syndrome (pSS), compared with AS (31).

With regard to platelet-derived eye drops, autologous PRP is still the most-used product for DED treatments, whereas allogeneic PRP, developed for other fields of application, is still in its early stages as an eye-drop treatment. As far as we know, these are the first cases of patients with DED due to SS treated with allogeneic PRP to be described in the literature. Further new data are needed need to confirm these very promising results.

## Conclusion

Allogeneic PRP applied topically to patients with SS-derived DED proved to be very effective in improving both symptoms and signs. PRP should not be understood as a replacement therapy for anti-inflammatory drugs, but as an adjunctive treatment to be combined with anti-inflammatory drugs in severe cases that are refractory to conventional therapies. Given its ability to improve corneal sensitivity, PRP eye drops could represent a treatment strategy that can be considered when nerve growth factor (NGF)-based eye drops are unavailable.

## Data availability statement

The original contributions presented in the study are included in the article/supplementary material. Further inquiries can be directed to the corresponding author.

## Ethics statement

Ethics committee approval is not required for this type of manuscript (Case series). The studies were conducted in accordance with the local legislation and institutional requirements. The participants provided their written informed consent to participate in this study. Written informed consent was obtained from the individual(s) for the publication of any potentially identifiable images or data included in this article.

## Author contributions

All authors listed have made a substantial, direct, and intellectual contribution to the work, and approved it for publication.
